# Improving Driver Alertness through Music Selection Using a Mobile EEG to Detect Brainwaves

**DOI:** 10.3390/s130708199

**Published:** 2013-06-26

**Authors:** Ning-Han Liu, Cheng-Yu Chiang, Hsiang-Ming Hsu

**Affiliations:** Department of Management Information System, National Pingtung University of Science & Technology, 1, Shuefu Road, Neipu, Pingtung 912, Taiwan; E-Mails: djmb3567@gmail.com (C.-Y.C.); one02piece@gmail.com (H.-M.H.)

**Keywords:** classifier, drowsy detection, EEG sensor, electroencephalogram, refreshing music

## Abstract

Driving safety has become a global topic of discussion with the recent development of the Smart Car concept. Many of the current car safety monitoring systems are based on image discrimination techniques, such as sensing the vehicle drifting from the main road, or changes in the driver's facial expressions. However, these techniques are either too simplistic or have a low success rate as image processing is easily affected by external factors, such as weather and illumination. We developed a drowsiness detection mechanism based on an electroencephalogram (EEG) reading collected from the driver with an off-the-shelf mobile sensor. This sensor employs wireless transmission technology and is suitable for wear by the driver of a vehicle. The following classification techniques were incorporated: Artificial Neural Networks, Support Vector Machine, and *k* Nearest Neighbor. These classifiers were integrated with integration functions after a genetic algorithm was first used to adjust the weighting for each classifier in the integration function. In addition, since past studies have shown effects of music on a person's state-of-mind, we propose a personalized music recommendation mechanism as a part of our system. Through the in-car stereo system, this music recommendation mechanism can help prevent a driver from becoming drowsy due to monotonous road conditions. Experimental results demonstrate the effectiveness of our proposed drowsiness detection method to determine a driver's state of mind, and the music recommendation system is therefore able to reduce drowsiness.

## 1. Introduction

Many accidents are caused by driver fatigue after driving for a prolonged period of time under monotonous road conditions. The occurrence of these accidents is likely to be lowered if warning signals are triggered when the driver becomes drowsy. In the past, various studies on the detection of driver fatigue involved installing devices on the vehicle to detect whether or not the vehicle had drifted within a short period of time, or the frequency at which the accelerator is being pressed. These methods can easily produce false detection events due to the driver's driving habits and road conditions. Other studies relied on capturing the frequency of eye movement as a reference for the level of drowsiness. However, detection results using this method could easily be compromised by external factors, for example the glare on the eye caused by external light sources would make the eye appear to be blinking. In order to more accurately determine the driver's state of mind, some researchers base their analysis directly on human physiological signals. Electroencephalography (EEG) is a device that can be applied to the analysis of human drowsiness. Although many methods are available for classifying drowsy brainwave patterns, the devices required are usually unsuitable to be installed inside a vehicle. Therefore, our study demonstrated the capture of brainwaves using well-developed and portable devices to be used to test the effectiveness of a number of common classification methods. Since each classification method has its own unique characteristics, we chose to integrate these classifiers in order to enhance the classification effectiveness.

There are five types of waves in an EEG defined by their frequency ranges [1]: the frequency of the α-waves fall between 8 and 13 Hz with amplitudes between 30 and 50 μV. A conscious person at rest in a quiet environment is likely to release α-waves from the parietal and the occipital regions of the brain. These waves disappear upon thinking or eye-blinking activities, or upon other types of stimulation. This phenomenon is called the alpha-block. The β-waves have frequencies between 14 and 30 Hz, and amplitudes between 5 and 20 μV; they usually appear in the frontal region of the brain while a person is conscious and alert, and are especially prominent when the person is thinking or undergoing sensory stimulation. The θ-waves have frequencies between 4 and 7 Hz and normally have amplitudes less than 30 μV. They are mainly released from the parietal and the temporal regions of the brain when a person is emotionally stressed, unconscious, or when the person's body is in a state of deep relaxation. The δ-waves have frequencies between 0.5 and 3 Hz, and amplitudes between 100 and 200 μV. A person will normally release δ-waves during deep sleep, when unconscious, deeply anesthetized, or if experiencing hypoxia. The δ-waves are almost never released when a person is conscious. The γ-waves have frequencies between 31 and 50 Hz with amplitudes ranging from 5 to 10 μV. Recent studies have found links between the γ-waves and selective attention, human cognition and perceptive activities.

All parts of the brain generate electrical signals, which are traditionally collected using the International 10-20 Electrode Placement System (10-20 System) by placing electrodes at 37 places on the scalp. This arrangement provides comprehensive monitoring of brainwaves, yet is inconvenient and impractical for a driver. Since human emotions, state of mind and focus are controlled by the frontal region of the brain, it is feasible to capture brain signals from only this area for the purpose of determining the drowsy state of a driver. In order to minimize inconveniences for the driver caused by wearing electrodes, we adopted an off-the-shelf portable wireless EEG device for the detection and analysis of signals generated by the frontal region of the brain.

Determining whether or not an EEG signal indicates drowsiness is a matter of classification. The most commonly adopted classifiers include Artificial Neural Network (ANN), Support Vector Machine (SVM) and *k* Nearest Neighbor Classifier (*k*NN). These three tools produce widely different results against the same set of data; in order to compensate for errors made by each tool and increase the classification accuracy, we consolidated and collectively processed all of the results. In addition, we used genetic algorithms to adjust the weighting of each of the classifiers in order to optimize the effectiveness of classification.

A driver will often use music to refresh themselves on a long-distance driving trip. Although there are a lot of mechanisms that can be used to refresh the drowsy driver, the in-car stereo system can achieve the goal with minimal cost. Furthermore, according to the experimental results in [2], when the subjects were listening to louder music when in a resting state, the drivers' autonomic nerve was activated. Therefore, we adopt music as the tool to refresh the driver. Our goal is to spontaneously play refreshing music upon the detection of drowsiness from the driver's brainwaves. Artificial intelligence is employed to analyze brainwaves released by the driver in response to various types of music and its content, and is able to select the appropriate music to play based on this data. The classification of brainwaves and music selection using this method has been experimentally proven.

The rest of this paper is organized as follows: Section 2 discusses relevant work. Section 3 describes the design of the electroencephalography classifiers. Section 4 includes the mechanism for refreshing the music recommendation. In Section 5 the experimental results are presented and discussed. The work concludes with Section 6.

## 2. Related Work

PERCLOS [3–5] is a driver fatigue measurement system based on the detection of ocular movement. Since PERCLOS employs image processing techniques, a strict environmental setting is required for accurate measurements. For example, strong external lighting would cause unnatural movements in the driver's eye, resulting in inaccurate detection. Recent research has shown new trends in fatigue measurement using physiological parameters. EEG has been widely applied in fatigue detection in studies relating to the central nervous system [6–8]. In [9], mental fatigue was evaluated using principal component analysis in conjunction with a cross-correlation function to compute the variation in the amplitude of the grand-averaged waveform at various points in time. The results demonstrated that the amplitude of the brainwaves would decrease as the level of mental fatigue increased. The study described in [10] looked at analyzing human consciousness from the EEG power spectrum, which would reflect changes in the level of alertness. The EEG power spectrum was paired with Independent Component Analysis (ICA) in [11]. The fast Fourier transform (FFT) was used to compute the brainwave spectrum, which was then used to calculate the level of fatigue. Lin *et al.* [12] established a sleepiness detection system, which determined the driver's cognitive state using statistical methods based on the analysis of multi-source EEG signals using ICA. The following study determined the level of fatigue by assessing the number of times the vehicle deviated from its course and the amount of time the driver needed to respond [13]. The study assumed that when the driver became drowsy the vehicle would deviate many times during a short interval. The study pointed out that when the driver began to take more time to correct the vehicle's drift, there were marked electrical activities in the occipital lobe, the posterior parietal lobe, and the middle temporal cortex. In addition, the power of α, δ, and θ frequency bands also increased, with the largest power increase in the α-waves. Complex installation procedures were required for the detection and recording equipment used in this study. In addition, the driver would find such a setup difficult to operate. Therefore, in our study light-weight equipment was adopted.

The studies in [14] and [15] captured the features of the EEG signals and used them as input to an ANN for the classification of brainwaves. In [16] and [17], a Discrete Wavelet Transform was used to analyze the EEG and the Daubechies 4 Wavelet Filter (DB4) was employed to categorize the signals into five levels. The features for these five levels were then fed into an ANN classifier. Another study used relative wavelet energy of the brainwaves as the input to the an ANN classifier [18], while [19] used Fast Independent Component (FastICA) to analyze the EEG before applying ANN classification.

A brainwave is a complex physiological signal and can be classified and processed using SVM. For example, [20] simplified brain image analysis by combining the Laplacian and SVM classifiers; in [21] a multiclass SVM with the capability to produce error-correcting output codes (ECOC) was used to classify EEG signals. The authors of [22] designed a Brain Computer Interface (BCI) based on analysis of brainwaves using SVM. This system identified brainwave signals, such as those indicating movement of the right or left hand, or carrying out simple mathematical calculations. A BCI system is constructed by a number of sensors placed in various places on the brain. However, the placement of the sensors in a BCI system would affect the results of classification. A Sensor Weighting SVM (sw-SVM) was proposed to cater for such effects [23].

The Dempster-Shafter theory (DS)-based *k*NN classifier was applied to EEG data collected in five different psychological events [24]. Experimental data showed that the DS-based *k*NN classification method produced considerably increased accuracy in comparison to the traditional *k*NN method. In addition, some studies focused on human EEG signals in response to stress, such as [25]. The authors classified the data using *k*NN and achieved an accuracy rate of 88.89%. Some studies adopted the Ensemble Learning method in order to enhance accurate classification of EEG signals. In [26], the authors attempted to identify the characteristics of EEG signals produced by humans during a learning experience using the *k*NN and Naïve Bayes classifiers. These were used in conjunction with the Self-Assessment Manikin (SAM) model that is used for determining emotions to classify and analyze the level of focus while learning.

In addition to using classifiers, brainwave signals can be classified based on mathematical and statistical models. Discriminative model and logistic regression techniques were used in [27] in the design of an EEG signal classification framework, which does not require the extraction of features or the removal of abnormalities from the EEG signals prior to classification. This still produces similar results to other classifiers. The study described in [28] proposed an algorithm for optimizing a spectral EEG filter; a common spatial pattern was incorporated in the design in order to increase classification accuracy. Another study proposed a Gaussian mixture model for the detection of neonatal seizure [29]. The study in [30] applied the partial-least squares algorithm to the real-time classification of brainwaves pertaining to the human cognitive state. This method remained robust against the influence of noise.

To help users find potential items of interest, recommendation systems are used. The most common types of recommendation systems utilize the content-based filtering method, the collaborative filtering method, or the hybrid recommendation method. The collaborative and hybrid filtering methods need the information provided by other members to predict a user's preference. We do not use the collaborative and the hybrid filtering methods since the EEG between users who listen to the same music is often different. A content-based filtering method characterizes and recommends items based on textual attributes, social tags, cultural information, and other kinds of web-based annotations. Music recommendation systems using the content-based filtering method extract the content features of songs, analyze these features, and then use them to compare the similarity of songs. This information is used to recommend new songs to users. For example, in [31,32] a user profile is created to store a few keywords and related information. This is performed in order to understand the user's music preferences and includes details such as the user's basic personal information, the types of music they like, and their favorite artists. The content attributes of each song can be regarded as different features, such as the name of the song, the singer, the genre, *etc.* The system will then find and recommend similar songs based on the user's profile. Pandora initiated The Music Genome Project [33] which attempts to analyze the content attributes of each song, such as melody, harmony, rhythm, musical instruments, and lyrics. A user only needs to input the names of the songs or artists that they desire and the system will find songs which are similar to recommend. A user can then rate the songs after listening to them. These ratings are used in the system as the basis for the next recommendation. The authors of [34] used cubic analysis of social tags to provide personalized music recommendations. In comparison, a high-level semantic description method was proposed in [35] to recommend music. The content-based technique was used in the research to automatically generate a semantic representation of the user's musical preferences directly from the audio.

## 3. Features Extraction and EEG Classification

**Figure 1.**
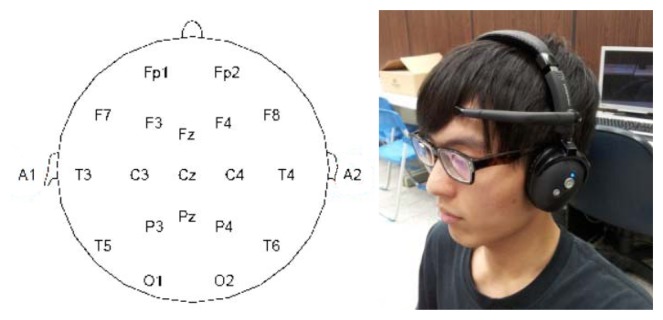
Brainwave collection points and a photo showing the device worn by a user.

We adopted the portable brainwave sensor by NeuroSky (San Jose, CA, USA). This device has the following properties: (1) easy to use, (2) safe and harmless, (3) dry electrode transducer, (4) wireless and portable, (5) capable of processing raw brainwave data, and (6) open platform with an API allowing for the development of related software applications on Android, iPhone, and DotNET platforms. This device collects brainwaves released from location FP1, as shown in Figure 1.

We extracted the EEG signals for each wave type from the driver's raw brainwave data and transformed them into respective features for classification. The real-time features were used as inputs for the classifier to evaluate the driver's level of drowsiness.

### 3.1. Features Extraction

The collected raw brainwave signals were processed with a discrete-time Fourier transform (DFT) before the power spectral density (PSD) was calculated. Normalized PSD values were used as features and input to various classifiers. The sample rate, Fs, used for data collection was 500 Hz. While there are six brainwave types, namely, α-waves, β-waves, δ-waves, θ-waves, γ-waves and μ-waves, only the former four types of waves are heavily relevant to human psychological states [11]. Therefore, in this study, we processed the brainwaves using a DFT and transferred the signals from the time domain to the frequency domain before the aforementioned four types of waves were transformed into frequency spectra. These frequency spectra are used in the following power calculations.

When the signal has been transferred from the time domain to the frequency domain, we can observe the signal's movements in each frequency range. We called the square of the coefficient of a spectrum's amplitude the spectrum's power spectral density (PSD) or spectral power distribution (SPD) [11]. Therefore, the post-DFT sampled signal sections could generate α-, β-, δ-, and θ- spectra for each sample section, respectively. The square of a brainwave's amplitude is the brainwave's power value. If we assume x(*t*′) is the sample series, where *t*′ = 0, 1, 2, …, 999, then the equation for PSD is:

(1) $$X(\omega )={\left|\text{FFT}(x(t'))\right|}^{2}$$

taking the sum of the PSD for each of α-, β-, δ-, and θ-waves over their respective frequency range would yield the brainwave's energy value for each frequency range:

(2) $$\begin{array}{l} \left\{ \begin{array}{ll} E_{\delta } & =\sum X(\omega ),0.5\leq f(i)\leq 3\\ E_{\theta } & =\sum X(\omega ),4\leq f(i)\leq 7\\ E_{\alpha } & =\sum X(\omega ),8\leq f(i)\leq 13\\ E_{\beta } & =\sum X(\omega ),14\leq f(i)\leq 30 \end{array}\right.\\ f(i)=\frac{f_{s}\times i}{T},i=1,2,3\cdots \frac{T}{2},T=1000 \end{array}$$

**Figure 2.**
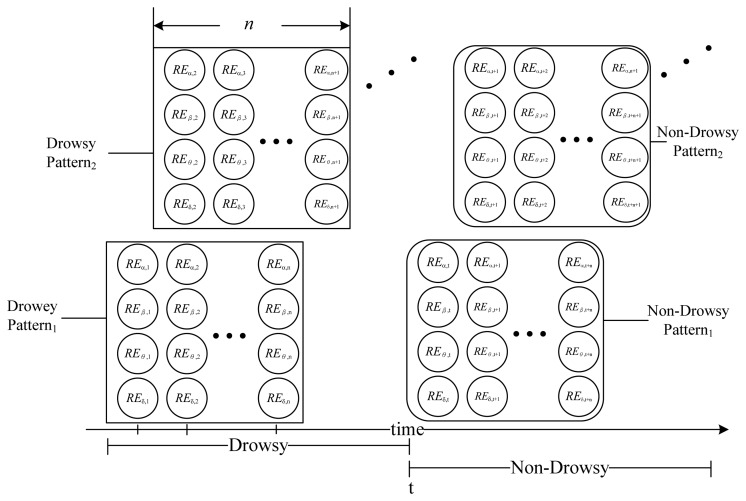
An example of EEG feature patterns.

For the PSD for each of α-, β-, δ-, and θ-waves at time *t* were denoted as *E*_α,t_, *E*_β,t_, *E*_δ_,*_t_* and *E*_θ,t_, respectively. Since a brainwave's signal strength differs from person to person, resulting in compromised classification accuracy due to different measuring reference points, we adopted the relative energy for each wave type. This was the ratio of energy in a frequency band to the total energy across all frequencies:

(3) $$\{\begin{array}{ll} RE_{\delta ,t} & =\frac{E_{\delta ,t}}{E_{\delta ,t}+E_{\theta ,t}+E_{\alpha ,t}+E_{\beta ,t}}\\ RE_{\theta ,t} & =\frac{E_{\theta ,t}}{E_{\delta ,t}+E_{\theta ,t}+E_{\alpha ,t}+E_{\beta ,t}}\\ RE_{\alpha ,t} & =\frac{E_{\alpha ,t}}{E_{\delta ,t}+E_{\theta ,t}+E_{\alpha ,t}+E_{\beta ,t}}\\ RE_{\beta ,t} & =\frac{E_{\alpha ,t}}{E_{\delta ,t}+E_{\theta ,t}+E_{\alpha ,t}+E_{\alpha ,t}} \end{array}$$

where *RE_δ,t_*, *RE_θ,t_*, *RE_α,t_* and *RE_β,t_* are the brainwaves' features. A brainwave signal is a time-based series therefore, we defined a pattern as a brainwave feature of length *n*, as shown in Figure 2.

Figure 2 shows that each pattern consists of 4×*n* values. We divided the raw data into two categories: Drowsy and non-drowsy, and the resultant patterns were to be the training data sets for the classifiers. The most effective pattern length is evaluated during experiments.

### 3.2. Mechanism of Drowsy State Classification

Sample data was fed to each classifier for training. Since each classifier classifies data differently, we consolidate results from all of the classifiers to create a classifier with an increased rate of accuracy. Once the classifiers have completed adjusting their parameters, they can begin processing real-time brainwave data, that is, determine whether or not the driver is drowsy.

#### • Classifier Based on Artificial Neural Networks (ANN)

**Figure 3.**
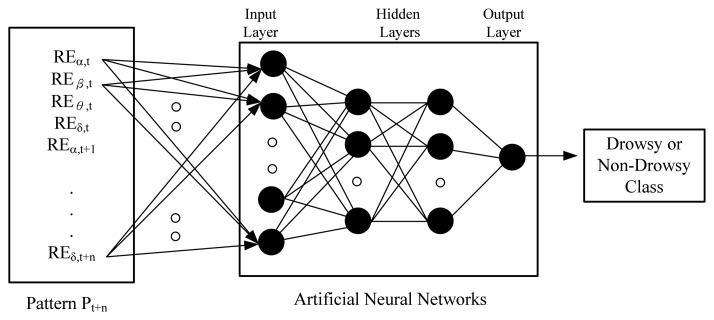
The structure of our artificial neural network.

ANN is a mature technology, which can be used in pattern classification. Having measured both types of pattern, the network starts to adjust weights between neurons via back-propagation [36]. In an ideal situation, the weights would converge and this completes the training. In a vehicle transport system, we collected the driver's EEG data in real-time and input their features to a trained neural network; the output from the neural network indicates if the driver was drowsy or non-drowsy. For example, if we marked the drowsy pattern with “+1” and the non-drowsy pattern with “0”, a trained ANN would be able to output a value between 0 and 1 from an input pattern. The closer the output is to 1, the more likely the driver is in the drowsy state, as shown in Figure 3. We evaluate the most appropriate number of layers for the ANN, since too many hidden layers creates an excessive calculation overhead.

#### • Classifier Based on a Support Vector Machine

**Figure 4.**
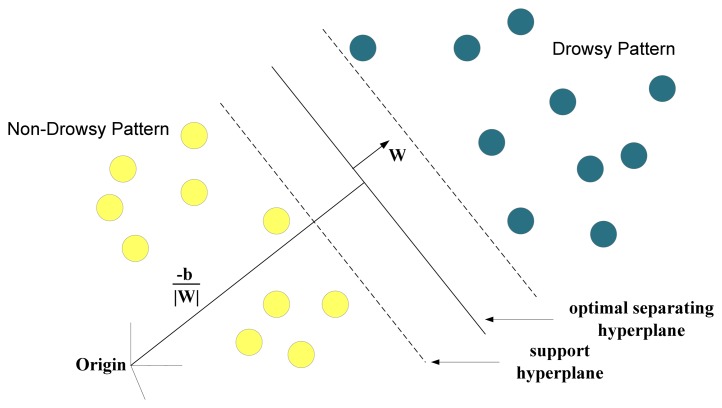
Illustration of SVM classification.

The idea of a SVM is to position a hyper plane to divide high-dimensional data into two separate sets, *i.e.*, classification [37]. Two hyper planes that separate two sets of data are parallel to each other and are closest to the training data point in their pertaining data set. The larger the distance between the parallel planes the smaller the classification error. Figure 4 illustrates the concept of SVM classification.

We used manually classified data patterns as training data for the SVM classifier. The training was deemed complete when a hyperplane was found such that the error was smaller than the value defined as the pre-condition. The SVM classifier would be able to determine if a real-time input pattern of the driver was a drowsy or a non-drowsy pattern. The classification result of the SVM was the probability of the input pattern being a drowsy pattern. If we assume the drowsy pattern is “1”, then the probability equation would be [38]:

(4) $$P(\text{Class}|\text{Input})=P(y=1|x)=\frac{1}{1+\exp(-f(x))}$$

where *f*(*x*) is the hyper plane of the SVM.

#### • *k*NN Classifier

The *k*-nearest neighbor (*k*NN) classifier is a kind of instance-based classifier that predicts the class of the unknown instances by relating the unknown to the known according to a distance function. We calculated the distance between the real-time brainwave pattern and other known patterns that had already been classified and selected *k* patterns that were the closest to each other. Within these *k* patterns, the ratio of drowsy patterns to *k* was the probability of the real-time brainwave that would indicate a drowsy state. The Euclidean distance was adopted to calculate the distance between patterns.

#### • Integrated Classification Method

The three aforementioned classification methods were sufficient to determine the drowsy state of a driver. It is instinctive to take the average of the three sets of results as the final outcome. However, we should adjust the weighting of each method since they do not yield the same accuracy. If we assume the drowsy state probability of a pattern is *P_1_* after ANN classification, *P_2_* after SVM classification, and *P_3_* after *k*NN classification, then the final probability *LP* after weighting adjustment is:

(5) $$\begin{array}{c} LP(\text{pattern})=\sum_{n=1}^{3}\left(w_{n}\times P_{n}\right)\\ \sum_{n=1}^{3}w_{n}=1,0\leq w_{n}\leq 1 \end{array}$$

where *w_1_*, *w_2_*, and *w_3_* are the respective weights of ANN, SVM, and *k*NN classifiers in the drowsy state calculation. We adopted a genetic algorithm to estimate the values for the weights since these calculations are non-trivial. A genetic algorithm is a search algorithm for optimization problems. The core theory behind genetic algorithms is to base the algorithm on observed traits from evolutionary biology, including inheritance, mutation, selection, and crossover [39].The steps of the genetic algorithm are:

##### Represent the Problem Domain as a Chromosome

**Figure 5.**
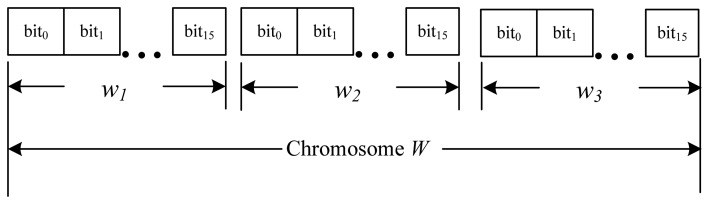
Data structure of chromosome.

For a weight vector *W*, we represent each weight value *w_n_* with 16 bits. The chromosome modeled is illustrated in Figure 5.

As 1 ≥ *w_n_* ≥0, the decoding function is defined as follows:

Assume the decimal value (base 10) of the 16 bits *w_n_* is (*D_n_*)_10_, then $w_{n}={\left(D_{n}\right)}_{10}\times \frac{1}{2^{16}}$.

##### Define a Fitness Function to Evaluate the Chromosome Performance

We aimed to adjust the values for *W* such that the total difference between the *LP* and the expected value for each training pattern would reach a minimum. For example: out of three patterns, pattern_1_ and pattern_2_ represented drowsy states with an expected *LP* of 1, and pattern_3_ represented a non-drowsy state with an expected *LP* of 0. If the actual *LP* values were 0.6, 0.9, and 0.2, respectively, the sum of differences would be | 1-0.6 | + | 1-0.8 | + | 0-0.2 | = 0.8. Assuming *Set_1_* was the set of drowsy patterns and *Set_2_* was the set of non-drowsy patterns, the sum of the difference between the training data and the expected values under the specific condition, *W*, could be defined as:

(6) $$\text{dif}(LP|{\text{Set}}_{1},{\text{Set}}_{2},W)\sum_{\text{pattern}\in {\text{Set}}_{1}}(1-LP(\text{pattern}))+\sum_{\text{pattern}\in {\text{Set}}_{2}}LP(\text{pattern})$$

The lower the difference, the higher the fitness value. Therefore, we define the fitness function as:

(7) $$f(W)=\frac{1}{\text{dif}(LP|{\text{Set}}_{1},{\text{Set}}_{2},W)+1}$$

##### Run the genetic algorithm and tune its parameters

The genetic algorithm achieves an acceptable value of *W* as follows [40]:

Step 1: We first randomly generate a population of chromosomes of size *S: W*_1_, *W*_2_, …, *W*_S_
Step 2: Next, we calculate the fitness of each chromosome: *f*(*W*_1_), *f*(*W*_2_), …, *f*(*W*_S_)
Step 3: A pair of chromosomes for mating are selected based on their fitness values.
Step 4: A pair of offspring chromosomes is created by applying the genetic operators: crossover with a probability *p_c_* and mutation with a probability *p_m_*.
Step 5: The resulting chromosomes are placed in the new population and step 4 is repeated until the size of the new chromosome population becomes equal to the size *S*.
Step 6: The current chromosome population is replaced with the new population.
Step 7: Return to step four and repeat the process until the termination criterion (e.g., number of generations) is met.

## 4. Refreshing Music Recommendation Mechanism

Past research has shown that music helps to increase alertness. In addition to developing a method to determine the level of driver fatigue, we also designed a music module which would automatically select refreshing music to play when the driver becomes tired from monotonous road conditions. Since the effects of music on a person's feelings and emotions differ from one individual to the next, the adopted artificial intelligence techniques were used to develop a music module that was able to deliver personalized music delivery services.

### 4.1. Music Features Extraction

As is widely known, music has characteristics including tempo and pitch. Traditionally, a digital music file can be either two formats: audio or symbolic data, for example, MIDI. The MIDI format is able to incorporate elaborate music styles and features and maintain an accurate representation of pitch and note length. We use the MIDI format to gather statistics of pitch and note length for use as features. Since the main melody is often indicative of the style of a piece of music, we extracted the following features [40]:

**Average Pitch (AP):** Indicates whether the piece of music is, on average, high pitched or low pitched:

(8) $$AP=\frac{\overset{n}{\underset{i=1}{\text{∑}}}v_{i}}{n}$$

where *v_i_* is the pitch scale of the note and *n* is the length of the sequence of notes.

**Pitch Entropy (PE)**: Indicates the degree of variation in the piece of music:

(9) $$PE=-\sum_{i=1}^{np}PN_{i}\log PN_{i}$$

where *np* is the number of distinct pitches that appear in the piece of music, and *PN_i_* is defined as:

(10) $$PN_{i}=\frac{Ni}{T}$$

where *N_i_* is the number of notes with the same pitch and *T* is the total number of notes.

**Pitch Density (PD):** Represents the number of different pitches that occur in the piece of music:

(11) $$PD=\frac{np}{128}$$

where *np* is the number of distinct pitches in the piece of music and 128 is the number of distinct pitches available in the MIDI standard.

**Average Duration (AD):** Describes the rhythm of the piece of music (for example, fast or slow):

(12) $$AD=\frac{\overset{n}{\underset{i=1}{\text{∑}}}D_{i}}{n}$$

where *D_i_* = *e_i_*-*s_i_*, is the duration of note and *n* is the length of the sequence of notes.

**Duration Entropy (DE):** Indicates the degree of variation between rhythms present in the piece of music:

(13) $$DE=-\sum_{i=1}^{nd}PR_{i}\log PR_{i}$$

where *nd* is the number of distinct durations that appeared in the piece of music and *PR_i_* is defined as:

(14) $$PR_{i}=\frac{Di}{T}$$

where *D_i_* is the number of notes with the same duration and *T* is the total number of notes.

**Pitch Interval Entropy (PIE):** Represents the degree of variation in the piece of music under key-invariant conditions:

(15) $$\text{PIE}=-\sum_{i=1}^{ni}PI_{i}\log PI_{i}$$

where *ni* is the number of distinct intervals that appear in the pitch interval string and *PI_i_* is defined as:

(16) $$PI_{i}=\frac{I_{i}}{TI}$$

where *I_i_* is the number of intervals with the same value and *TI* is the length of the interval string.

While the features obtained from the MIDI files were used to classify music, our music database contained music in both MIDI and Wave formats. Wave music files were used in the vehicle's stereo system.

### 4.2. Music Classifier

To each individual, music can be divided into two types: refreshing and non-refreshing. We were able to determine whether or not a piece of music appeared refreshing to a driver by examining the driver's brainwaves at the time the music is being played, that is, the disappearance of drowsy brainwaves would indicate that the music currently playing is refreshing to this particular driver. The following table of data was collected after the system was utilized for a length of time.

Table 1 allows us to determine the features that would make a piece of music appear refreshing to a particular driver. This system uses the C4.5 algorithm [41] for dealing with continuous numerical attributes for category learning as the basis to build up the user's refreshing music model. The C4.5 algorithm is used to generate a decision tree. The decision tree generated by C4.5 can be used for classification. We will discuss how to establish the user's refreshing music model in each step by employing this algorithm [33].

Step 1: **Interval Initialization**
  The order of the values in a feature, *F*, from small to large, e.g., *F* = {*A*_1_, *A*_2_, …, *A*_n_}, allows us to establish a candidate cut point, *CP*. This point is obtained through the calculation between adjacent numbers, defined as:
  (17) $$CP=(A_{i}+A_{i+1})/2$$
  where *A_i_* stands for the continuous numerical values within the interval, *A_i_*_+1_ is the adjacent number of *A_i_*, and *CP* is the value of the cut point.
Step 2: **Search the Real Cut Point Position**
  A candidate cut point is obtained through the search and used to calculate the maximum information gain in order to determine the position of the real cut point. The maximum information gain is calculated as the ‘entropy before cut’ minus the “entropy after cut”, this is expressed as:
  (18) $$\text{Informatio nGain}\;\text{(}F\text{)}={\text{Entropy}}_{\text{before}}\;(S)-{\text{Entropy}}_{\text{after}}\;(S)$$
  If it is required to calculate the information gain of *F*, the entropy after the cut is needed, such as that shown in Equation (19). When *n* results are included in *S* incidents, *Cp* is the probability of each result corresponding to a type. The entropy in *S* is:
  (19) $${\text{Entropy}}_{\text{after}}\;(S)=\sum_{k=1}^{n}-C_{p}\log_{2}C_{p}$$
  which is used to calculate the comparison between the cut point and information gain, and determine a point *K* as the position of the real cut point. The real cut point can be used to divide the numerical attribute information into two sections, “≤ *K*” and “> *K*”, by means of binary segmentation.
Step 3: **Select the Best Features of the Attribute Nodes**
  The results after segmentation are passed to the gain ratio function to calculate the importance of this attribute. If an attribute *F* is included in *S*, this calculation is needed. This is calculated as the “entropy before cut ‘minus’ entropy after cut”, and then divided by the “entropy after segmentation”, as:
  (20) $$\text{GainRatio}(F)=\frac{{\text{Entropy}}_{\text{before}}(S)-{\text{Entropy}}_{\text{after}}(S)}{{\text{Entropy}}_{\text{after}}(S)}$$

Various features are applied to calculate and compare with the gain ratio; the larger value will be chosen to be considered as the root node. We are able to determine the next feature node and branch by recursive repetition of Steps 1 to 3, until either every subset belongs to the same category, or there is no information to be used for classification.

**Figure 6.**
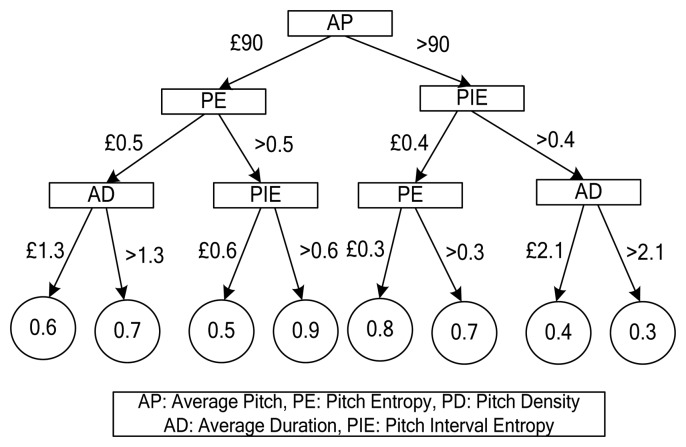
An example of a user's refreshing music decision tree.

A leaf node in a decision tree represents the category (refreshing or non-refreshing) of the pieces of music that satisfy the conditions of a decision. We limit the height of the decision tree to eliminate problems of over fitting. A leaf node in a segregated decision tree may contain both types of music; therefore we used the ratio of each type of music against the total number of music pieces, as classes. Figure 6 shows an example of a refreshing music decision tree.

There are four levels in the decision tree shown in Figure 6. The number in a leaf node is the refreshing score with a value between 0 and 1; it represents the ratio of the number of refreshing music pieces against the total number of music pieces. For example, a song with the following attribute values AP = 40, PE = 0.3 and AD = 0.9 is classified as 0.6 by the decision tree, which means the refreshing effectiveness of this song is 0.6.

### 4.3. Music Scheduling Mechanism

A piece of music was given a refreshing score (*i.e.*, classes in the decision tree) by the refreshing music decision tree. However, having the system play the music piece with the highest refreshing score may result in a single piece of music being selected every time refreshing music needs to be played. Our initial experiments and observation revealed that the user lost interest in repeating music, possibly resulting in reduced refreshing effectiveness for that particular piece of music. Therefore, to avoid playing just the highest scoring song every time, we use the scores as the probability of playing each song. The higher the score, the higher the probability for the song to be played. In other words, for a music set *C*, if song *s* has a score of *F*(*s*), then the probability that *s* is played is:

(21) $$P(s)=\frac{F(s)}{\underset{s'\in C}{\text{∑}}F(s')}$$

## 5. Experiments

There were two primary sections to our experiment. First, the coefficients of the ANN, SVM, and *k*NN classifiers were adjusted to optimize classification efficiency. Second, tests were carried out to evaluate if the integration of the three classifiers would result in increased classification accuracy.

### 5.1. Experimental Results of Drowsy Patterns Classification

**Figure 7.**
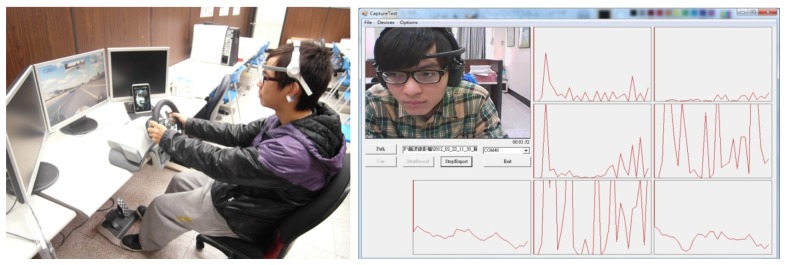
A driving simulation system for the collection of driver's brainwaves.

Since it is difficult to collect a large number of EEG patterns during an actual driving experience, our test subject used a driving simulation system to simulate driving on a freeway at night. The system set up is shown in Figure 7. To accurately identify the states of the subject's EEG signals, in addition to recording the EEG signal data during the experimentation process, the facial image at the time of the experiment was also recorded through a camera. The test subject's state of mind (drowsy or non-drowsy) was manually determined by observing brainwaves and video collected by the simulation system. In the other words, if the video clips show that the test object was drowsy, the brain patterns for the associated time segment were labeled as drowsy patterns. In contrast, the brain patterns were labeled as non-drowsy patterns for the inverse case. The test subjects included 20 males and 20 females between the ages of 20 and 25. 600 brainwave patterns consisting of 300 drowsy patterns and 300 non-drowsy patterns were collected from each test subject. The *k*-fold cross validation method was used to evaluate the efficiency of the classifiers. During testing for each classifier we can determine the optimum EEG pattern length (*PL*).

The energy of each type of brainwave was also used as a feature in addition to *RE* defined earlier. The correctness ratio as a result of classification using these features is called the *RE-Correctness* when using *RE* as features and *E-Correctness* when using the energy as features. *Correctness* is defined as:

(22) $$\text{Correctness}=\frac{\text{Number of correctly classified patterns}}{\text{Number of classified patterns}}\times 100\%$$

A false negative error, *i.e.*, when an actual drowsy state brainwave is classified as a non-drowsy state, is considered a critical error as it will result in the system not prompting the driver at all. A false negative error (*FN*) is defined as:

(23) $$FN=\frac{\text{Number of drowsy patterns classified as non}-\text{drowsy state}}{\text{Number of drowsy patterns}}\times 100\%$$

False negative errors when classifying using *RE* as features are named *RE-FN*, and *E-FN* when classifying using energy as features. A false alarm occurs when a non-drowsy state is classified as a drowsy state – a false positive error:

(24) $$FP=\frac{\text{Number of non}-\text{drowsy patterns classified as drowsy state}}{\text{Number of non}-\text{drowsy patterns}}\times 100\%$$

False positive errors when classifying using *RE* as features are named *RE-FP*, and *E-FP* when classifying using energy as features. We desire the system in our design to have less *FN* than *FP* in order to reduce the risk of accidents due to a non-reported drowsy state.

#### Analysis of ANN Training Experiments

Since the number of hidden layers in an ANN has an effect on the system's efficiency, it was important to select an appropriate number of layers. Our goal was to achieve optimal classification accuracy with a relatively small number of layers. In this experiment, the number of neurons in each layer was set to be twice as many as the dimensions of the pattern. The output layer was set to 1 as there were only two classes. The results are shown in Table 2. As shown in Table 2, increased accuracy was achieved when the pattern length (*PL*) was greater than 3, while similar results were obtained when *PL* = 4. If we consider the speed of the ANN calculations, we chose patterns of length 3 to be the inputs to the classifier. In addition, the classification accuracy increased with patterns that used *RE* as features than those that used energy as features, which indicates that *RE* features were more suitable for determining a drowsy state. ANNs that used *RE* patterns produced very similar precision when the number of hidden layers was three, four, and five. On the other hand, more *RE-FP* than *RE-FN* incidents were produced when the number of hidden layers was three. Therefore, the number of hidden layers for the consolidated ANN was set to three, taking into consideration both the execution speed and accuracy.

#### Analysis of the SVM Training Experiments

As different kernel functions produce different classification outcomes, it is crucial to choose a suitable kernel function for an SVM [37]. Commonly used kernel functions include linear functions, polynomial functions, radial basis functions, and sigmoid functions. The same data used for the ANN experiments was also used here to test the classification efficiency of various kernel functions. The results are listed in Table 3.

Table 3 shows that the highest accuracy was achieved with a *PL* of 3. Therefore, as with the ANN classifier, patterns of length 3 were used for the SVM classifier. The table also indicates the highest accuracy when the SVM classifier used the polynomial kernel functions and *RE* features. The difference between the occurrences of *RE-FN* and *RE-FP* was small. We adopted the polynomial functions as the kernel functions for the SVM classifier for the system.

#### Analysis of the kNN Training Experiments

We conducted analysis on the value of *k* to be used in a *k*NN classifier as it would have an impact on the accuracy of the classification. We adopted the class in which most of the *k* closest patterns fell to be the class for testing the patterns, hence an odd number for the value of *k*. The experimental results for the *k*NN classifier are listed in Table 4.

We can see from Table 4 that the classification accuracy was the highest when *PL* = 4, hence the EEG pattern length of 4 for the *k*NN classifier. The value of *k* was set as 3 since the best classification accuracy was achieved when *k* = 3 and the value of *RE-FP* was the smallest when *RE* features were used. If we compare the results listed in Tables 2, 3 and 4 we can see that the SVM classifier produced the best classification results while the *k*NN classifier had the worst accuracy. However, the *k*NN classifier produced the best *RE-FN* outcomes. Therefore, in the following experiments we employ genetic algorithms to adjust the weighting and consolidate the results from all three classifiers.

#### Analysis of the Integrated Classifier Experiment

In our previous experimental results an unsatisfactory classification outcome when energy was used as features was seen, and therefore, energy as a feature is excluded from this experiment in which the three classifiers are integrated. The best values of coefficients for all three classifiers from previous experiments are also used for this experiment. In this experiment, the probability of crossover was 0.7 and the probability of mutation was 0.001. Table 5 shows the results of the impact of varying number of generations on the accuracy of the classification.

We can see, from the results listed in Table 5 that an integrated classifier would achieve better classification results because: (1) classification accuracy reached a stable state once the number of generations reached 3,000, (2) classification accuracy of the *RE-Correctness* was better than that from each of the three classifiers independently, and (3) the number of *RE-FN* incidents decreased.

### 5.2. Experimental Results of Refreshing Music Scheduling

In this stage, we evaluated the effectiveness of the machine-picked refreshing music to reducing drowsiness. The experiment involved ten test subjects consisting of five males and five females, and 100 pieces of music in the music database. Each subject has one personal refreshing music decision tree. A different test subject would spend a long period of time each day at the same driving simulator used earlier in this study. The test subjects' EEG data was collected while music was being played. A music recommendation would be regarded as successful when the refreshing music stopped a drowsy EEG pattern from occurring. While no drowsy pattern was detected, the test subjects could make their own, or the system would play a random, selection of music. A test subject had their own dedicated decision tree, which was updated once a week. The criteria for evaluating the effectiveness of the refreshing music scheduling is:

(25) $$\text{Precision}=\frac{\text{Count of Successful Recommendations}}{\text{Total Number of Recommendations}}\times 100\%$$

**Figure 8.**
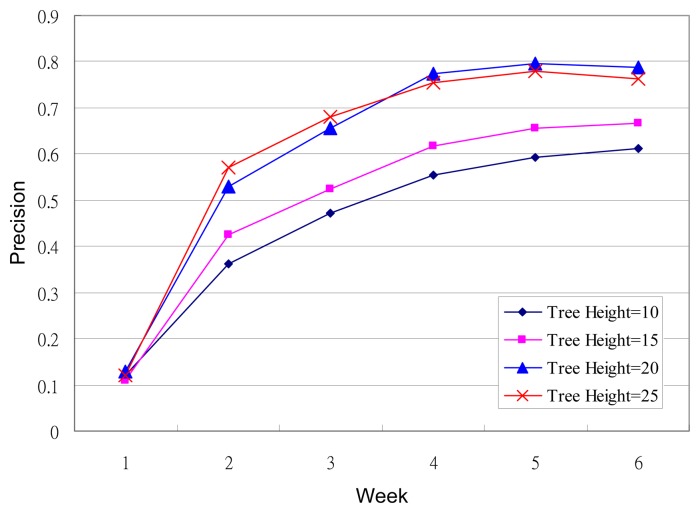
Experimental results for the refreshing music recommendations.

The experiment was conducted over six weeks and the results are shown in Figure 8. Random music was played during the first week due to a lack of data to construct a decision tree. The system was able to make better music recommendations as the decision came to effect in week two. As can be seen, the precision value began to stabilize from week five. The experimental data shows that the precision grew with the height of the decision tree before the Tree Height reached 25, after which the Tree Height had little effect on the precision. Overall, a success rate of close to 80% can be achieved based on the EEG data collected over the weeks.

## 6. Conclusions

As EEG devices become smaller and more portable, it is possible to help refresh and alert a drowsy driver using the driver's state of mind, which can be determined from their brainwave data. In this work, we proved through experiments, that brainwave features and discriminating patterns can be used to determine whether or not the driver is in a drowsy state. The classification effectiveness of the ANN, SVM, and *k*NN classifiers were examined. These classifiers were eventually integrated using integration functions after a genetic algorithm was used to adjust the weighting for each classification method in the integration function. The integrated classification function achieved better performance than the individual classifiers alone. The system we designed did not only detect the driver's state of mind but also incorporated a mechanism for recommending refreshing music. Music selection was based on learnt experiences from the user's brainwave data. Experimental results were seen to produce positive outcomes for the driver.

All experiments were carried out using a driving simulation system for safety reasons; an extension of our study will be to conduct experiments using actual vehicles to further evaluate the effectiveness of our system in reducing a driver's drowsiness. In order to improve the accuracy of drowsiness detection we aim in future studies to consolidate our system with image processing techniques to detect the frequency of the opening and closing of the driver's eyes. This is a commonly adopted method for drowsiness detection. Many sensor devices for human physiological changes are being miniaturized, for example, blood oxygen and heart rate sensors. These devices can potentially be integrated with our system and help increase the accuracy of the classification. In our experiments, the training samples of drowsy and non-drowsy patterns were subjectively defined. Therefore, we will incorporate brain specialists or researchers to identify the patterns objectively in the future.

## Figures and Tables

**Table 1.** An example of a user table. *F*_1_, *F*_2_, …, *F*_6_ are the music features.

**Music ID**	**F**_**1**_	**F**_**2**_	**F**_**3**_	**F**_**4**_	**F**_**5**_	**F**_**6**_	**Refreshing Music**
5	82	0.9	0.5	1.3	0.4	0.3	Yes
9	75	0.4	0.3	2.1	0.3	0.2	No
13	64	0.6	0.1	0.5	0.2	0.5	Yes

**Table 2.** Experimental results of ANN classifier (%).

**Pattern Length (PL)**	**Hidden Layer**	**RE-Correctness**	**RE-FP**	**RE-FN**	**E-Correctness**	**E-FP**	**E-FN**
1	1	55.9	38.7	49.3	47.1	49.7	56.1
	2	60.5	36.3	42.7	49.2	48.8	52.8
	3	66.2	35.9	31.8	50.1	48.9	50.9
	4	66.1	31.9	35.9	51.1	46.9	50.9
	5	66.2	30.5	37.2	51.1	49.9	48.0
2	1	57.2	36.8	48.8	48.3	47.2	56.2
	2	62.6	34.4	40.4	50.5	46.5	52.5
	3	69.0	31.6	30.3	52.2	45.9	49.7
	4	69.2	30.2	31.4	53.2	45.9	47.8
	5	69.2	29.0	32.7	53.4	44.5	48.6
**3**	1	61.2	34.9	42.7	52.4	43.8	51.4
	2	65.9	32.1	36.1	53.9	42.4	49.8
	**3**	**72.4**	**28.7**	**26.5**	53.2	45.9	47.8
	4	72.5	26.4	28.6	54.3	43.0	48.4
	5	71.9	26.2	29.9	52.6	49.3	45.5
4	1	58.5	38.2	44.8	59.3	35.0	46.4
	2	64.8	33.8	36.6	51.7	44.4	52.2
	3	72.4	29.3	26.0	54.4	41.9	49.2
	4	72.3	26.6	28.8	55.1	42.3	47.6
	5	72.1	25.7	30.2	55.6	42.6	46.1

**Table 3.** Experimental results of SVM classifier (%).

**Pattern Length (PL)**	**Kernel**	**RE-Correctness**	**RE-FP**	**RE-FN**	**E-Correctness**	**E-FP**	**E-FN**
1	Linear	62.60	38.15	36.65	51.90	49.06	47.14
	Polynomial	69.10	32.14	29.66	54.30	48.44	42.96
	Radial basis	65.60	36.46	32.34	51.58	50.36	46.48
	Sigmoid	66.50	34.84	32.16	52.01	47.03	48.95
2	Linear	66.90	32.44	33.76	56.10	43.02	44.78
	Polynomial	72.30	29.92	25.48	58.80	42.85	39.55
	Radial basis	69.10	32.14	29.66	55.48	45.41	43.63
	Sigmoid	70.10	30.50	29.30	54.81	42.48	47.90
**3**	Linear	69.3	31.9	29.4	58.4	43.2	40.0
	**Polynomial**	**75.3**	**25.2**	**24.2**	60.2	40.6	39.0
	Radial basis	71.8	29.3	27.1	57.9	44.6	39.6
	Sigmoid	73.2	28..4	25.2	58.2	45.1	38.4
4	Linear	68.20	33.07	30.53	57.30	40.99	44.41
	Polynomial	73.20	26.26	27.34	59.70	42.72	37.88
	Radial basis	71.70	29.43	27.17	57.58	44.12	40.72
	Sigmoid	72.20	28.36	27.24	57.11	41.17	44.61

**Table 4.** Experimental results of *k*NN classifier (%).

**Pattern Length (PL)**	**k**	**RE-Correctness**	**RE-FP**	**RE-FN**	**E-Correctness**	**E-FP**	**E-FN**
1	1	49.70	76.46	24.14	49.70	63.38	37.22
	3	51.50	72.75	24.25	51.10	70.42	27.38
	5	50.20	75.70	23.90	50.20	66.73	32.87
	7	49.80	74.30	26.10	50.40	60.51	38.69
	9	50.10	69.86	29.94	49.70	54.32	46.28
2	1	50.52	74.22	24.74	49.90	72.14	28.06
	3	52.25	70.67	24.83	51.63	66.75	29.99
	5	51.14	71.34	26.38	50.66	71.05	27.63
	7	50.59	78.07	20.75	51.07	62.63	35.23
	9	50.73	71.93	26.61	50.63	58.26	40.48
3	1	52.34	69.58	25.74	51.13	61.58	36.16
	3	54.10	66.10	25.70	53.03	67.64	26.30
	5	54.08	66.12	25.72	51.64	64.80	31.92
	7	52.38	69.53	25.71	52.49	57.96	37.06
	9	52.56	70.21	24.67	51.49	52.39	44.63
4	1	53.2	77.7	15.9	52.2	65.0	30.6
	**3**	**55.6**	**74.6**	**14.2**	53.4	64.3	28.9
	5	54.8	67.8	22.6	52.6	61.6	33.2
	7	53.5	67.8	25.1	52.5	53.2	41.8
	9	53.7	63.0	29.6	52.3	50.6	44.8
5	1	52.99	76.16	17.86	51.94	66.32	29.80
	3	55.29	70.64	18.78	53.05	66.67	27.23
	5	54.63	66.24	24.50	51.85	65.48	30.82
	7	53.30	67.25	26.15	51.91	54.82	41.36
	9	53.40	64.31	28.89	51.61	54.20	42.58

**Table 5.** Experimental results of integrated classifier (%).

**Generations**	**RE-Correctness**	**RE-FP**	**RE-FN**
1000	76.3	28.9	18.5
2000	77.4	29.3	15.8
**3000**	**81.3**	**23.9**	**13.5**
4000	81.2	24.3	13.3
